# Physical Exercise Preserves Adult Visual Plasticity in Mice and Restores it after a Stroke in the Somatosensory Cortex

**DOI:** 10.3389/fnagi.2016.00212

**Published:** 2016-09-21

**Authors:** Evgenia Kalogeraki, Justyna Pielecka-Fortuna, Janika M. Hüppe, Siegrid Löwel

**Affiliations:** ^1^Department of Systems Neuroscience, JFB Institut für Zoologie und Anthropologie, Georg-August Universität GöttingenGöttingen, Germany; ^2^Göttingen Graduate School for Neurosciences, Biophysics, and Molecular Biosciences (GGNB)Göttingen, Germany

**Keywords:** adult plasticity, visual cortex, stroke, physical exercise, ocular dominance

## Abstract

The primary visual cortex (V1) is widely used to study brain plasticity, which is not only crucial for normal brain function, such as learning and memory, but also for recovery after brain injuries such as stroke. In standard cage (SC) raised mice, experience-dependent ocular dominance (OD) plasticity in V1 declines with age and is compromised by a lesion in adjacent and distant cortical regions. In contrast, mice raised in an enriched environment (EE), exhibit lifelong OD plasticity and are protected from losing OD plasticity after a stroke-lesion in the somatosensory cortex. Since SC mice with an access to a running wheel (RW) displayed preserved OD plasticity during aging, we investigated whether physical exercise might also provide a plasticity promoting effect after a cortical stroke. To this end, we tested if adult RW-raised mice preserved OD plasticity after stroke and also if short-term running after stroke restored OD plasticity to SC mice. Indeed, unlike mice without a RW, adult RW mice continued to show OD plasticity even after stroke, and a 2 weeks RW experience after stroke already restored lost OD plasticity. Additionally, the experience-enabled increase of the spatial frequency and contrast threshold of the optomotor reflex of the open eye, normally lost after a stroke, was restored in both groups of RW mice. Our data suggest that physical exercise alone can not only preserve visual plasticity into old age, but also restore it after a cortical stroke.

## Introduction

Neuronal plasticity in the adult brain is indispensable to allow adaptive changes during aging and after lesions. A well-known model system to study experience-dependent circuit changes is ocular dominance (OD) plasticity in the primary visual cortex (V1): occluding one eye of vision for a couple of days induces an OD shift towards the open eye. This phenomenon was first described in kittens (Wiesel and Hubel, 1963) and later in mice (Dräger, 1978). OD-plasticity is age-dependent: in mice, it is most pronounced in juvenile animals (postnatal day (P) 28), reduced in young adults (P90) and absent beyond P110 if mice are raised in standard cages (SCs; Lehmann and Löwel, 2008; Espinosa and Stryker, 2012). Interestingly, raising or exposing aged rodents to an enriched environment (EE), with increased physical (running wheels (RWs)), social (bigger housing groups) and cognitive (regularly changed labyrinths or toys) stimulation, can overcome plasticity limitations (Sale et al., 2007; Baroncelli et al., 2010; Scali et al., 2012) and preserve a lifelong OD-plasticity in mice (Greifzu et al., 2014, 2016a). Surprisingly, just one of these components has also been recently shown to preserve plasticity into older age in mice: adult mice raised in SCs with a RW continued to show OD-plasticity beyond P134, while age-matched SC-mice (without a RW) did not exhibit this plasticity (Kalogeraki et al., 2014). Furthermore, even short-term running, just during the 7-day MD period also restored OD-plasticity to adult SC-raised mice.

Plasticity is not only important for normal brain function and during aging but also for recovery after brain injuries like stroke; understanding plasticity mechanism is therefore essential for further development of adequate therapies. We have previously shown that a small stroke lesion in the primary somatosensory cortex (S1) prevented OD-plasticity in V1 of adult SC-raised mice (Greifzu et al., 2011). Remarkably, raising mice in EE protected adult mice from lesion-induced impairments of visual plasticity, similarly as young mice were protected: EE-mice continued to display OD-plasticity even after stroke, suggesting that EE preserves a more juvenile brain into adulthood (Greifzu et al., 2014). These observations implicated that young and/or physically active animals in a cognitively and socially challenging environment are less affected by a distant brain lesion and preserved their visual cortical plasticity even in adulthood. Whether all of these components are necessary for a plasticity promoting effect after stroke or just one of them has not yet been tested. Because both long-term and short-term voluntary physical exercise promoted OD-plasticity in aging mice (Kalogeraki et al., 2014), here we tested whether running alone might already provide a beneficial effect on visual plasticity after a cortical lesion in S1. We focused on running because locomotion has recently been shown to massively increase pyramidal cell firing in V1 (Niell and Stryker, 2010; Fu et al., 2014) and promote activity-dependent changes in V1 circuitry (Kaneko and Stryker, 2014). Do adult mice with access to a RW (in an otherwise SC) continue to exhibit OD-plasticity in V1 even after an S1-stroke? And if so, is long-term running required for preserving OD-plasticity or is it possible to restore OD-plasticity in adult SC-raised mice even *after* stroke by putting them in cages with a RW? Our present data clearly show that voluntary running was effective in both experimental conditions: running preserved OD-plasticity after stroke both in adult mice with access to a RW for all their life and in animals that started running only after their stroke lesion. Thus, voluntary physical exercise may help to prevent plasticity impairments after a cortical lesion and in addition, might be a useful noninvasive therapy to support rehabilitation in an early post-stroke period.

## Materials and Methods

### Animals and Rearing Conditions

All mice were from the mouse colony of the central animal facility, University Medical Center Göttingen, and housed with a 12 h light/dark cycle, with food and water available *ad libitum*. All experimental procedures were approved by the local government (Niedersächsisches Landesamt für Verbraucherschutz und Lebensmittelsicherheit, registration number 33.9-42502-04-10/0326). The experimental procedures comply with National Institutes of Health guidelines for the use of animals. The cages were translucent with an open grid cover and wood chip bedding.

#### Long-Term Running Groups (RW-mice)

Pregnant C57BL/6J females were put into slightly larger than normal SCs (27 × 43 × 19 cm) equipped with a RW (RW-cage) 6–11 days before delivery. The offsprings were separated into female and male groups at postnatal day (P) 28. A total number of 17 male and female mice between P149 and P222 were used for this study. Male and female mice were mixed in these groups because previous publications indicated that there is no statistical difference between the values of female and male mice of a similar age for all of the measured parameters: ocular dominance index (ODI), V1-activation, spatial frequency and contrast sensitivity thresholds (e.g., Kalogeraki et al., 2014). Mice were randomly assigned to experimental groups and were housed in a group of 3–5 animals per cage.

#### Short-Term Running Groups (14dRW-mice)

Mice were raised in SCs (26 × 20 × 14 cm) without a RW until at least P119, and transferred to RW-cages (size see above) after induction of the lesion. Eighteen male mice born and raised in SCs were used (age range: P119–258). Since most of the animals of this group were from different litters, mice had to be housed alone in the RW-cages to avoid fighting. The number of RW-turns for every mouse was counted daily.

After monocular deprivation (MD)/noMD, spatial vision was checked daily by optomotry for 7 days. Finally, we visualized V1-activation after stimulation of the ipsi- and contralateral eye using intrinsic signal optical imaging (Cang et al., 2005).

### Induction of a Photothrombotic (PT) Stroke

All animals were at least P119 before PT/sham-treatment. The model of photothrombosis (PT) was used to induce a cortical lesion in the left S1 using the Rose Bengal technique (Watson et al., 1985) as described previously (Greifzu et al., 2011). PT is a minimally invasive technique to induce localized and small lesions restricted to the cortex. Briefly, mice were box-anesthetized with 2% isoflurane in a mixture of O_2_/N_2_O (1:1), the body temperature was maintained at 37°C via a heating pad and the animals’ head was placed in a stereotaxic frame. The skin above the skull was incised and an optic fiber bundle (aperture: 1.0 mm), mounted on a cold light source (Schott KL 1500, Germany), was positioned 2 mm lateral to the midline and 1 mm posterior to the bregma. Hundred microliters Rose Bengal dye (0.1% in saline) was injected intravenously into the tail vein. After 5 min of waiting time, the illumination of the cortex started for 15 min. Finally the skin was sutured and animals returned to their home cages for recovery. Lesion size was determined at the end of the behavioral and optical imaging experiments by Glia Fibrillary Acidic Protein (GFAP) staining. For the controls, the exact same procedure was followed without turning on the light source (RW-sham).

### Monocular Deprivation (MD)

The right eye of the mice was deprived for 7 days, according to published protocols (Gordon and Stryker, 1996; Cang et al., 2005; Lehmann and Löwel, 2008). Briefly, mice were box anesthetized with 2% isoflurane in O_2_/N_2_O (1:1), eyelids were trimmed and closed with two sutures. Mice were returned to their home cages for recovery and checked daily to ensure that the eye remained closed. In case of an open MD-eye, mice were excluded from further experiments.

### Virtual-Reality Optomotor Setup

Both the spatial frequency threshold (“visual acuity”) and the contrast threshold (“contrast sensitivity”) of the optomotor reflex of all mice (animals with and without MD) were measured using the virtual-reality optomotor system (Prusky et al., 2004). Briefly, freely moving mice were positioned on a small platform surrounded by four computer monitors (33.5 × 26.5 cm) forming a box. Mirrors were placed at the bottom and the top of the box, giving the impression to the animal that it was sitting in an endless cylinder. A rotating virtual cylinder, composed of a vertical sine wave grating, was projected on the screens. Parameters like spatial frequency, contrast and speed of the moving sine wave grating could be varied by the experimenter. In case the mouse could detect the stimulus, it was reflexively tracking the grating by moving the head in the direction of rotation. Spatial frequency thresholds at full contrast and contrast thresholds at six different spatial frequencies [0.031, 0.064, 0.092, 0.103, 0.192, 0.272 cycles/degree (cyc/deg)] were measured daily (after MD/no MD) for this study.

### Optical Imaging of Intrinsic Signals and Visual Stimuli

After completion of all behavioral vision tests, visual cortical responses were recorded and analyzed as described previously (Greifzu et al., 2014; Kalogeraki et al., 2014).

#### Surgery

Briefly, mice were box-anesthetized with 2% halothane in O_2_/N_2_O (1:1) and injected with atropine (0.1 mg/mouse s.c.; Franz Köhler), dexamethasone (0.2 mg/mouse s.c.; Ratiopharm), and chlorprothixene (0.2 mg/mouse i.m.; Sigma). After placing mice in a stereotaxic frame, anesthesia was maintained with 0.8% halothane in a 1:1 mixture of O_2_/N_2_O. An incision of the skin was made over the visual cortex and low-melting point agarose (2.5% in 0.9% NaCl) and a glass coverslip were placed over the exposed area.

#### Data Acquisition and Visual Stimulation

Mouse V1-responses were recorded through the skull using the Fourier imaging technique (Kalatsky and Stryker, 2003), optimized for the assessment of OD-plasticity (Cang et al., 2005). V1-signals were visualized with a CCD-camera (Dalsa^®^ 1 M30) using a 135 × 50 mm tandem lens configuration (Nikon), with red illumination light (610 ± 10 nm). Active brain regions absorb more of the red light and appear darker in the images. Frames were acquired at a rate of 30 Hz, temporally binned to 7.5 Hz and stored as 512 × 512 pixel images after spatial binning of the camera image.

Visual stimuli were presented on a high refresh rate monitor (Hitachi, ACCUVUE, HM-4921-D, 21″) positioned 25 cm from the eyes. Stimuli consisted of white drifting horizontal bars (2° wide). The distance between two bars was 70° and they were presented at a temporal frequency of 0.125 Hz. To calculate OD, the visual stimulus was restricted to the binocular visual field of the left V1 (−5° to +15° azimuth, 0° azimuth corresponding to frontal direction) and animals were stimulated through either the left or the right eye in alternation.

#### Data Analysis

Visual cortical maps were calculated from the acquired frames by Fourier analysis to extract the signal at the stimulation frequency using custom software (Kalatsky and Stryker, 2003). While the phase component of the signal is used for the calculation of retinotopy, the amplitude component represents the intensity of neuronal activation (expressed as the fractional change in reflectance ×10^−4^) and was used to calculate OD. At least 3 maps per animal were averaged to compute the ODI as follows: (C − I)/(C + I), with C and I representing the response magnitudes of each pixel to visual stimulation of the contralateral and ipsilateral eye. The ODI ranges from −1 to +1, with negative values representing ipsilateral and positive values representing contralateral dominance. For comparison, we used previously published data of PT-lesioned C57Bl6/J (WT) mice without access to a RW (presented in Figures 3, 7): ODI-data of PT-lesioned (S1) late MD WT mice (MD started 1 week after PT = SC-PT (1w)) are from Greifzu et al. (2011), but newly analyzed as described above (“average map analysis”). In addition, SC-PT groups (noMD and MD) are taken from Greifzu et al. (2016b).

### Lesion Analysis

To determine the size and location of the cortical PT-lesions, coronal brain sections were cut at 40 μm, and immunostaining with an antibody against GFAP was performed. GFAP is a common marker used to reveal brain lesions (Lai et al., 2014) by staining the astrocytes that are accumulating at the lesion site. Lesions were visible in all mice that received a PT-stroke. Sections were washed for 10 min with 0.1 M PB and incubated for 10 min with 0.1 M PB-Triton-X-100 (0.3%), followed by 30 min blocking in 10% normal donkey serum in PB-Triton-X-100 (0.3%) at room temperature. The sections were incubated with the primary polyclonal rabbit-anti-GFAP antibody (Immunological Science) 1:1000, diluted in 0.1 M PB-Triton-X-100 (0.3%) over night at 4°C. The following day the sections were washed 3 times for 5 min with 0.1 M PB. Incubation with the secondary Cy3-goat-anti-rabbit antibody (Jackson ImmunoResearch Inc.) was for 2 h at room temperature in dark (1:1000 diluted in PB-Triton-X-100 (0.3%)), followed by three washes for 5 min with 0.1 M PB. Sections were then transferred onto microscope slides and mounted with Fluoromount-G with 4′6-diamidin-2-phenylindol (DAPI; Jackson ImmunoResearch Inc.). For quantitative lesion analyses, every third section was analyzed under the microscope (Axioskop, Carl Zeiss) using AxioVision (AxioVs 40 4.5.0.0.). To quantify the position of each lesion, the distance from the center of the lesion to the midline and also to the anterior border of V1 were measured. In addition, we determined lesion length (anterior-posterior), diameter (medial-lateral), depth and calculated the total volume of each lesion.

### Statistical Analyses

All intra- and intergroup comparisons were analyzed either by a two-tailed *t*-test or one-way analysis of variance (ANOVA) followed by multiple comparisons Bonferroni correction. The intergroup comparison of the enhancement of the spatial frequency and contrast sensitivity thresholds was analyzed by a two-way ANOVA. The levels of significance were set as *p < 0.05; **p < 0.01; ***p < 0.001. Data are represented as means ± SEM.

## Results

### Location and Size of the Cortical Lesion in the Primary Somatosensory Cortex (S1)

**Figure 1:**
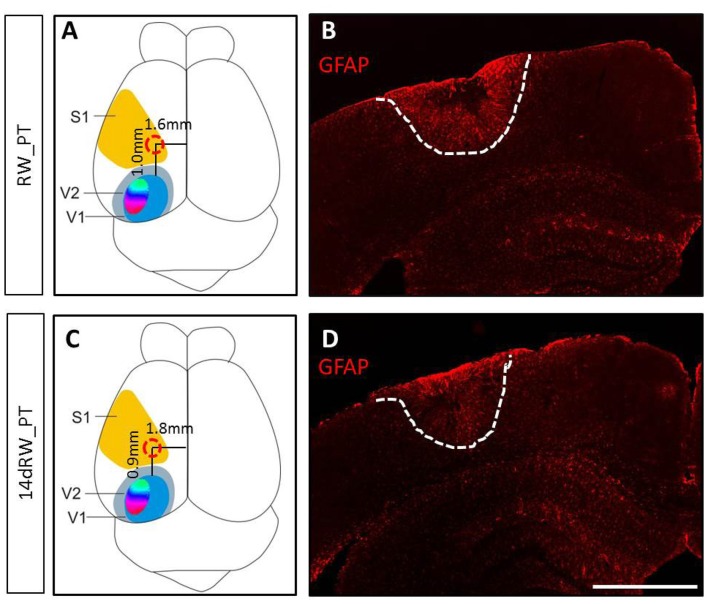
**Location and size of the photothrombotically induced cortical stroke lesion in primary somatosensory cortex (S1) of both long-term (A,B) and 14dRW-mice (C,D).** **(A,C)** Schematic representation of a mouse brain illustrating the average lesion location in S1 (red circle) for running wheel (RW)-photothrombotic (PT) mice **(A)** and 14dRW-PT mice **(C)**. Abbreviation: primary/secondary visual cortex = V1/V2. **(B,D)** Glia Fibrillary Acidic Protein (GFAP)-stained frontal section through the lesion. Lesion borders are marked with a white dashed line. Scale bar: 1 mm.

The PT-lesions were always located in the left S1 (Figure 1). Both lesion location and volume were not different between the lifelong running (RW-PT) and the short-term running mice (14dRW-PT; *p* > 0.05, for each parameter, *t*-test): the lesion center was located 1.0 ± 0.2/0.95 ± 0.2 mm anterior to the anterior border of V1 and 1.6 ± 0.1/1.8 ± 0.1 mm lateral to the midline for the RW-mice and 14dRW-mice, respectively. Average lesion volume was 2.75 ± 0.9 for RW-PT and 2.04 ± 0.4 mm^3^ for 14dRW-PT-mice (*p* = 0.40, *t-test*). In detail, the size of the lesion in RW-PT mice was on average 1.2 ± 0.2 mm in medio-lateral, 1.4 ± 0.2 mm in anterio-posterior direction and extended vertically until layers 5/6 (*n* = 7). For the 14dRW-PT mice, the lesion was on average 0.8 ± 0.1 mm in medio-lateral, 1.2 ± 0.1 mm in anterio-posterior direction and also extended until layers 5/6 (*n* = 12). Notably, volume and location of the present PT-lesions were not different from the PT-lesions of SC-mice without RWs (Greifzu et al., 2011; *p* > 0.05 for all measured parameters) which showed abolished visual plasticity.

### Voluntary Physical Exercise Preserved OD-Plasticity in Adult RW-Mice After Stroke

#### Analyses of V1-Activity Maps in the Lesioned Hemisphere

**Figure 2:**
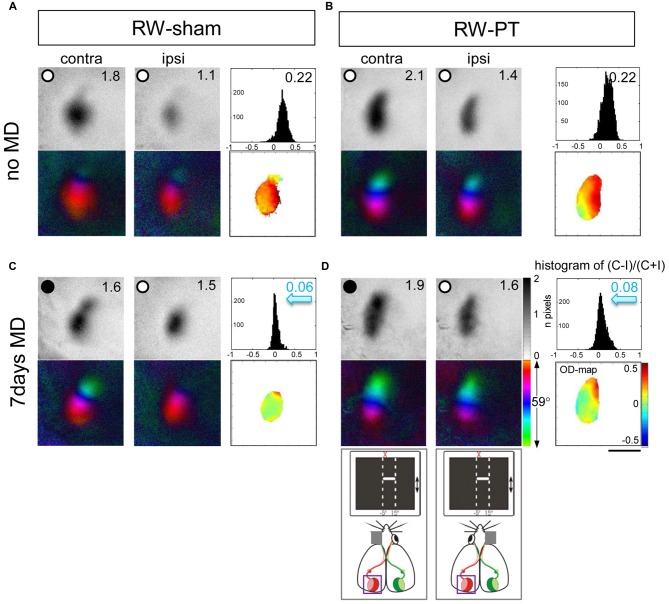
**Running promoted ocular dominance (OD)-plasticity in adult (>P149) mouse V1 even after stroke in the adjacent S1.** Optically recorded activity and retinotopic maps of the contralateral (contra) and ipsilateral (ipsi) eye in the binocular part of V1 of non-lesioned (running wheel (RW)-sham; **A,C**) and PT-lesioned mice raised in standard cages (SCs) with a RW (RW-PT; **B,D**). Gray-scale coded activity maps (numbers correspond to quantified V1-activation), the histogram of OD-scores, including the average ocular dominance index (ODI), 2-dimensional OD-maps and color-coded polar maps of retinotopy are illustrated. In mice without MD **(A,B)**, activity patches evoked by visual stimulation of the contralateral eye are darker than those of the ipsilateral eye, ODI-values are positive and warm colors prevail in the 2-dimensional OD-maps. Seven days of monocular deprivation (MD; MD-eye is indicated by the black spot) resulted in a strong OD-shift in both the lesioned and non-lesioned groups (sham: **C**, PT: **D**), both eyes activated binocular part of V1 equally strong; the ODI values were lower, the OD-histograms shifted to the left (blue arrows) and colder colors prevailed in the OD-maps. Scale bar: 1 mm.

**Figure 3:**
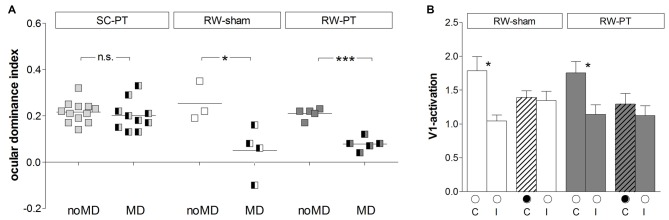
**Quantification of V1-activation in both PT- and sham-lesioned mice: running preserved OD-plasticity in V1. (A)** Optically imaged ODIs of PT-lesioned SC-mice (left, light gray), RW-sham (middle, white) and RW-PT mice (right, gray) without and with MD. Symbols represent ODI-values of individuals; means are marked by horizontal lines. MD is indicated by half-black squares. In contrast to SC-mice, MD induced an OD-shift in RW-mice regardless of the treatment. **(B)** V1-activation elicited by stimulation of the contralateral (C) or ipsilateral (I) eye in RW-sham and RW-PT mice without and with MD. Black filled circle indicates MD eye. *p < 0.05, ***p < 0.001.

It was previously reported that a small cortical lesion in the S1 impaired visual plasticity of SC-raised mice (Greifzu et al., 2011). In contrast, the same cortical lesion did not compromise plasticity in mice raised in an EE (Greifzu et al., 2014), pointing out that active and stimulating environments have protective effects for brain injuries and may promote recovery. As already one of the components of EE, voluntary physical exercise preserved OD-plasticity into late adulthood (Kalogeraki et al., 2014) here we investigated if free access to a RW would preserve OD-plasticity also after an S1 lesion. Indeed, we found that mice raised in SCs with a RW continued to display OD-plasticity into adulthood, both in sham- and in PT-animals (Figures 2, 3), which is in contrast to SC mice with no access to a RW (Figure 3). Without MD, V1 of both the RW-sham (control, Figure 2A) and RW-PT mice (Figure 2B) was dominated by the contralateral eye: activity patches induced by visual stimulation of the contralateral eye were darker than those after stimulation of the ipsilateral eye, the average ODI-values were positive and warm color dominated the 2-dimensioned OD-maps (Figures 2A,B). After 7 days of MD, both adult RW-sham (>P156) and RW-PT mice (>P149) displayed OD-shifts towards the open eye: the binocular part of V1 was activated more similarly after visual stimulation of the contra- or ipsilateral eye, the OD-histogram was shifted to the left (ODI-values were closer to zero), and colder colors appeared in the 2-dimensional OD-maps (Figures 2C,D). The preserved OD-shift in adult RW-sham mice confirmed our recent finding of preserved OD-plasticity in aging RW-mice (Kalogeraki et al., 2014). Notably, visual cortical maps of adult RW-PT mice were rather indistinguishable from the maps of RW-sham mice. Thus, RW-mice not only continued to display OD-plasticity into a much older age compared to SC-mice, they additionally showed OD-plasticity even after a cortical stroke, unlike even younger SC-mice without access to a RW (Greifzu et al., 2011).

Quantitative analysis of V1-activation revealed that both groups of RW-mice displayed an OD-shift towards the open eye after MD, independent of the presence of a cortical lesion (Figure 3A). In the RW-sham group, the ODI decreased from 0.25 ± 0.05 (*n* = 3, P180–215) to 0.05 ± 0.05 after MD (*n* = 4, P156–218; *p* = 0.04, *t*-test). Similarly, in RW-PT mice, the ODI decreased from 0.21 ± 0.01 (*n* = 5; P174–222) to 0.08 ± 0.01 after MD (*n* = 5, P149–204; *p* < 0.0001, *t-test*). Differences between sham- and PT-lesioned mice with MD were not significant (*p* > 0.05, ANOVA). Also, mice without MD from all groups had similar mean ODI-values (*p* > 0.05, ANOVA).

Analyzing V1-activation induced by left and right eye stimulation after MD revealed that in both groups of RW-mice V1 was nearly equally activated by both eyes (Figure 3B). Compared to the no-MD condition V1-activation via the deprived (contralateral) eye was lower after MD, but the reductions in deprived eye responses in V1 were not significant. In detail, V1-activation after contralateral eye stimulation was 1.79 ± 0.20 in RW-sham mice and reduced to 1.40 ± 0.10 after MD (*p* = 0.116, *t-test*). In RW-PT mice V1-activation was 1.76 ± 0.17 and reduced to 1.30 ± 0.16 after MD (*p* = 0.076, *t-test*). In RW-sham mice ipsilateral eye activation of V1 was 1.04 ± 0.09 and 1.35 ± 0.14 after MD (*p* = 0.139, *t-test*). In RW-PT mice ipsilateral eye activation of V1 was 1.14 ± 0.14 and 1.12 ± 0.15 after MD (*p* = 0.931, *t-test*).

#### Analyses of V1-Maps in the Non-Lesioned (Right) Hemisphere (Figure 4)

To stimulate the binocular part of the right V1 a moving horizontal bar was positioned to −15 to +5 degrees of the left visual field. In this case, the deprived (right) eye was ipsilateral and the open (left) eye contralateral to the imaged hemisphere. After 7 days of MD, V1-activition after ipsilateral (deprived) eye stimulation was reduced, resulting in an even stronger contralateral dominance. The average ODI-value of RW-sham mice increased from 0.21 ± 0.02 (*n* = 3) without MD to 0.29 ± 0.02 (*n* = 3) after MD. Likewise, in RW-PT mice, the ODI-value increased from 0.23 ± 0.04 (*n* = 5) to 0.29 ± 0.03 after MD (*n* = 4). Although the ODIs were higher after MD in both groups, the differences were not significant (*p* = 0.064/0.357, *t*-test).

**Figure 4:**
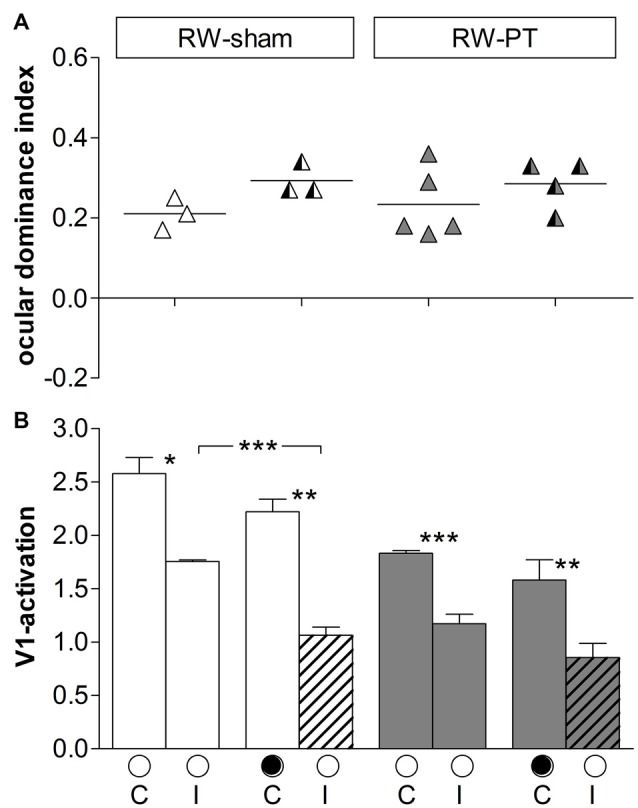
**ODIs and V1-activation in V1 of the non-lesioned right hemisphere in RW-mice after PT or sham treatment. (A)** Optically imaged ODIs without and with MD of sham treated (RW-sham, white) and PT-mice (RW-PT, gray) raised in RW-cages. Symbols represent ODI-values of individuals; means are marked by horizontal lines. **(B)** V1-activation elicited by stimulation of the contralateral (C) or ipsilateral (I) eye without and after MD. Data are displayed as in Figure 3. *p < 0.05, **p < 0.01, ***p < 0.001.

Quantitative analysis revealed that in RW-sham mice, V1-activation via the deprived (ipsilateral) eye was 1.75 ± 0.01 and reduced to 1.06 ± 0.08 after MD (*p* = 0.009, *t-test*); in contrast, V1-activation after contralateral eye stimulation was unchanged (noMD/MD: 2.58 ± 0.15/2.22 ± 0.12, *p* = 0.14, *t-test*). In the RW-PT mice, V1-activation after ipsilateral eye stimulation was 1.17 ± 0.09, and 0.86 ± 0.13 after MD (*p* = 0.07, *t-test*). After contralateral eye stimulation in RW-PT mice, no significant difference was observed in V1-activation (noMD/MD: 1.83 ± 0.02/1.58 ± 0.19; *p* = 0.18, *t-test*).

### Running Preserved the Experience-Enabled Enhancement of the Optomotor Reflex of the Open Eye After a Cortical Stroke in S1

In SC raised mice, a stroke in S1 completely abolished improvements of the optomotor reflex of the open eye after MD (Greifzu et al., 2011). Here we tested spatial vision (spatial frequency and contrast thresholds) of all RW-mice before and after MD using the virtual-reality optomotor setup (Prusky et al., 2004).

#### Spatial Frequency Threshold

The highest spatial frequency that elicited an optomotor reflex in the RW-mice measured before MD and PT was 0.38 ± 0.001 cyc/deg for RW-sham mice (*n* = 7) and 0.38 ± 0.001 cyc/deg for RW-PT mice (*n* = 10). Baseline values were thus not different between the groups (*p* > 0.05, ANOVA).

**Figure 5:**
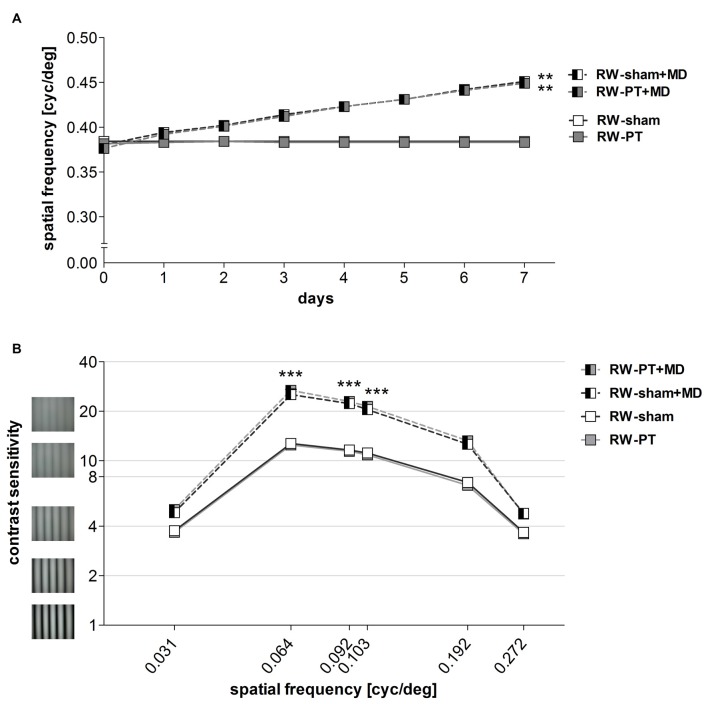
**Enhanced optomotor reflex after MD in RW-mice. (A)**The spatial frequency threshold of the optomotor reflex in cyc/deg plotted as a function of days. **(B)** Contrast sensitivity thresholds of optomotor reflex measured on 7th day of MD/noMD in six different spatial frequencies. All the groups without MD are illustrated as boxes and with MD as half-filled boxes. PT-groups are presented in gray color and sham groups in white. Note that there was a significant improvement of thresholds after 7 days of MD regardless of the induction of a lesion. There was no significant difference between sham- and PT-mice after MD, nor between sham- and PT-mice without MD. **p < 0.01, ***p < 0.001.

While values without MD remained stable (*p* > 0.05, for both groups, ANOVA), daily testing of both groups of RW-mice *after* MD resulted in an increase of the measured values, i.e., an increase of the highest spatial frequency grating that elicited an optomotor reflex in both non-lesioned and PT-lesioned mice: in RW-sham mice, the spatial frequency threshold of the optomotor reflex of the open eye increased to 0.45 ± 0.002 cyc/deg (*n* = 4) and to 0.45 ± 0.002 cyc/deg in RW-PT mice (*n* = 5; Figure 5A). Values on day 7 after MD were different from day 0 for both groups (*p* < 0.01, ANOVA). The increase in spatial frequency thresholds was similar for both groups after MD and equal to 23% for RW-sham and 23% for RW-PT mice (*p* > 0.05, ANOVA). Values between non- and PT-lesioned RW-mice were indistinguishable (*p* > 0.05 for both comparisons; ANOVA).

#### Contrast Sensitivity Threshold

Furthermore, contrast sensitivity thresholds of the optomotor reflex were determined at six different spatial frequencies (0.031, 0.064, 0.092, 0.103, 0.192 and 0.272 cyc/deg) for all the groups (Figure 5B). Baseline (before MD) contrast sensitivity values were not different between RW-sham and RW-PT mice (*p* > 0.05; for every frequency, ANOVA). Additionally, values were similar to values previously described for C57BL/6J mice (Prusky et al., 2004; Lehmann and Löwel, 2008).

After 7 days of MD, contrast sensitivity of the optomotor reflex of the open eye increased (i.e., the lowest contrast that elicited an optomotor reflex decreased) in both RW-sham and RW-PT mice (*p* < 0.001, ANOVA): thresholds increased significantly for 3 out of the 6 spatial frequencies measured after MD (at 0.031 cyc/deg: sham/PT: to 4.8 ± 0.1 (corresponds to 21% contrast)/5.0 ± 0.2 (20%), 0.064 cyc/deg: 26.2 ± 1.7 (4%)/27.0 ± 1.2 (4%), 0.092 cyc/deg: 23.1 ± 1.4 (4%)/23.4 ± 0.9 (4%), 0.103 cyc/deg: 21.2 ± 1.2 (5%)/21.3 ± 0.5 (5%), 0.192 cyc/deg: 12.6 ± 0.5 (8%)/ 12.9 ± 0.6 (8%), 0.272 cyc/deg: 4.8 ± 0.1 (21%)/4.7 ± 0.1 (21%) on day 7; *p* > 0.05, *p* < 0.001, *p* < 0.001, *p* < 0.001, *p* > 0.05, *p* > 0.05, compared to values from day 0, ANOVA). There were no differences between the two RW-groups at any spatial frequency on day 7 (*p* > 0.05, ANOVA).

Thus, in contrast to PT-lesioned SC-raised mice (Greifzu et al., 2011), experience-enabled enhancements of the optomotor reflex of the open eye after MD were present and thus preserved after stroke in mice raised in cages with a RW.

### Voluntary Physical Exercise *After* Stroke Rescued OD-Plasticity in Adult Mice (Figure 6)

In addition to long-term running, we also tested the therapeutic potential of short-term voluntary physical exercise for stroke recovery. To this end, we raised mice in normal SCs until at least P119, the age limit for OD-plasticity of SC-mice (Lehmann and Löwel, 2008), and then induced a PT-lesion in S1 as before. Immediately after the PT-surgery, both sham- and PT-treated mice were transferred to a SC with a RW (14d RW-mice). When we performed PT and MD on the same day most animals were running only very little in the first 4 days after PT + MD (on average 0.4 ± 0.02 km/day/mouse), and running values increased only thereafter to values of about 2.0 ± 0.4 km/day/mouse (similar to previously published values; Kalogeraki et al., 2014); thus we decided to provide mice with a recovery time of 7 days after PT before we initiated MD.

**Figure 6:**
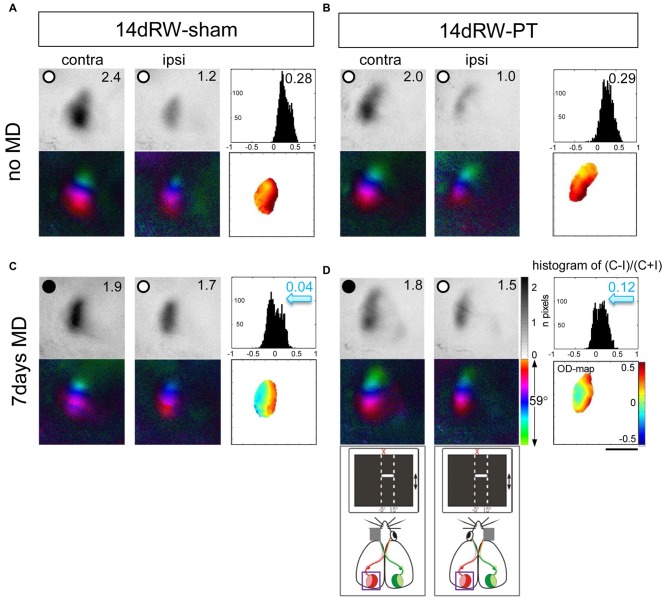
**Short-term running *after* an S1-stroke restored OD-plasticity in adult (>P119) mouse V1.** Optically recorded maps of the contralateral (contra) and ipsilateral (ipsi) eye of non-lesioned (14dRW-sham: **A,C**) and PT-lesioned 14dRW mice raised in a SC but transferred to a RW-cage *after* stroke (14dRW-PT; **B,D**), Data presented as in Figure 2. Activity and polar maps of the binocular part of the left V1 and ODI values of the left hemisphere, 2-dimensional OD-maps and ODI-histograms without **(A,B)** or with MD **(C,D)**. Note that animals with MD showed a significant OD-shift in both groups of 14dRW-mice (blue arrow indicates reduced ODIs), i.e., OD-plasticity was restored in adult mice by 14dRW-experience regardless of the treatment (sham or PT). Scale bar: 1 mm.

As in SC-raised C57Bl/6J mice, V1 of control 14dRW-mice (both 14dRW-sham and 14dRW-PT) was dominated by input from the contralateral eye: activity maps induced by visual stimulation of the contralateral eye were darker than those after ipsilateral eye stimulation, the ODI had positive values and the 2-dimensional OD-map was dominated by warm colors (Figures 6A,B). After MD, contra- and ipsilateral eyes activated V1 equally strong in both groups of 14dRW-mice: activity maps after contra- and ipsilateral eye stimulation were rather similarly dark, ODI values were reduced, and the ODI-histogram was shifted to the left compared to the no-MD condition (control), and colder colors appeared in the 2-dimensional OD-maps (Figures 6C,D).

**Figure 7:**
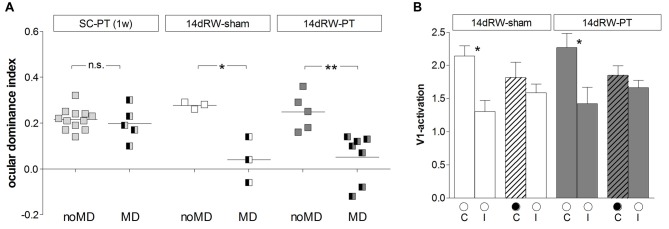
**Quantification of ODIs and V1-activation: short-term running *after* stroke restored OD-plasticity in V1 of adult SC-mice.** **(A)** Optically imaged ODIs without (noMD) and with MD of SC-raised PT-lesioned mice (SC-PT; light-gray, left), non-lesioned 14dRW-mice (14dRW-sham; white, middle) and PT-lesioned 14dRW-mice (14dRW-PT; dark gray, right). Data displayed as in Figure 3. In contrast to SC-PT mice, animals of both 14dRW-groups showed an OD-shift after MD regardless of the treatment (sham/PT). **(B)** V1-activation elicited by stimulation of the contralateral (C) or ipsilateral (I) eye without and with MD in non- and PT-lesioned 14dRW-mice. *p < 0.05, **p < 0.01.

Quantitative analysis of V1-activation confirmed that also adult short-term RW-mice showed an OD-shift after MD, irrespective of whether they had a lesion in S1 (14dRW-PT) or not (14dRW-sham; Figure 7). In contrast to SC-raised PT-lesioned mice (SC-PT; Figure 7A, left), adult mice (>P119) continued to display OD-plasticity even in the presence of a cortical stroke (in S1) if they had access to a RW for just 14 days *after* stroke induction (Figure 7A, middle and right). In effect, 14dRW-PT mice showed OD-plasticity of a similar magnitude as age-matched adult 14dRW-sham mice: the ODI of the 14dRW-sham group decreased from 0.28 ± 0.09 (*n* = 3, P119–162) to 0.04 ± 0.06 after MD (*n* = 3, P120–197; *p* = 0.012, *t-test*). Likewise, the ODI of the 14dRW-PT group decreased from 0.25 ± 0.04 (*n* = 5, P124–258) to 0.05 ± 0.04 after MD (*n* = 7, P119–213; *p* = 0.006, *t-test*). Differences between 14dRW-mice (sham vs. PT) in all conditions were not significant (*p* > 0.05, ANOVA). Since in a previous report, a waiting period of 1 week between PT and a 7-day-MD did not restore OD-plasticity after stroke (Greifzu et al., 2011), the restoration of OD-plasticity in the 14dRW-mice must be due to the new running experience and cannot be due to the one-week delay between PT and MD.

We additionally quantified V1-activity maps after stimulation of each eye (Figure 7B). In 14dRW-sham mice, V1-activation after contralateral eye stimulation was 2.14 ± 0.15 and slightly but not significantly reduced to 1.81 ± 0.23 after MD (*p* = 0.305, *t*-test); after ipsilateral eye stimulation V1-activation was 1.30 ± 0.17 and 1.59 ± 0.13 after MD (*p* = 0.249, *t-test*). In 14dRW-PT mice, V1-activation after contralateral eye stimulation was also non-significantly reduced from 2.27 ± 0.21 to 1.85 ± 0.14 after MD (*p* = 0.119, *t-test*), V1-activation after ipsilateral eye stimulation was 1.42 ± 0.25, and 1.66 ± 0.11 after MD (*p* = 0.344, *t-test*).

#### OD-Plasticity in the Non-Lesioned Hemisphere of 14dRW-Mice

**Figure 8:**
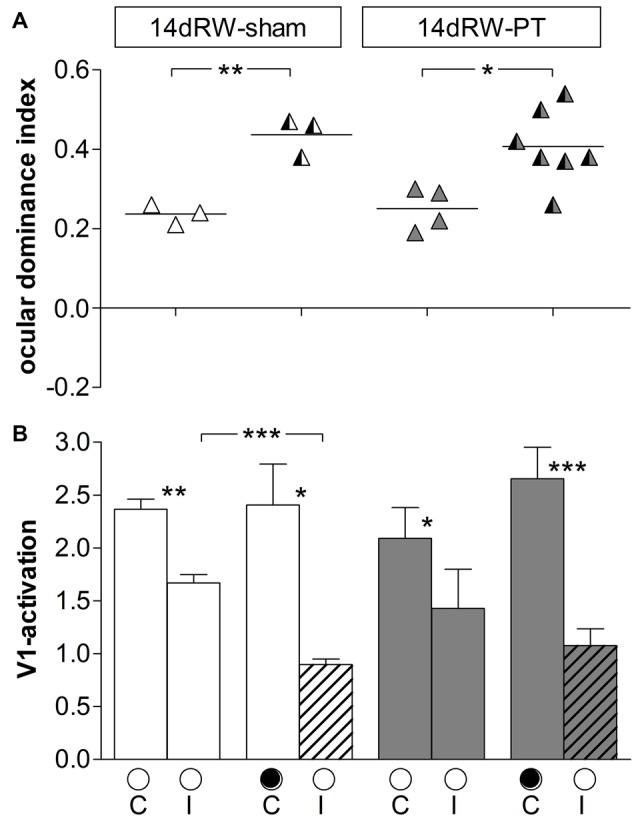
**ODIs and V1-activation in V1 of the non-lesioned (right) hemisphere of non-lesioned and PT-lesioned 14dRW-mice. (A)** Optically imaged ODIs without and with MD of 14dRW-sham (white) and 14dRW-PT mice (dark-gray). Data displayed as in Figure 3. **(B)** V1-activation elicited by stimulation of the contralateral (C) or ipsilateral (I) eye without and after MD. *p < 0.05, **p < 0.01, ***p < 0.001.

In the right (non-lesioned) hemisphere, the ODI of both 14dRW-sham and 14dRW-PT-mice increased significantly after MD (Figure 8): for sham/PT 14dRW-mice, the average ODI was 0.24 ± 0.01/0.25 ± 0.03 (*n* = 3/4), and increased to 0.44 ± 0.03/0.40 ± 0.03 after MD (*n* = 3/7; *p* = 0.003/0.031, *t*-test).

In 14dRW-sham mice, V1-activation after visual stimulation of the previously deprived (ipsilateral) eye was significantly reduced from 1.67 ± 0.08 to 0.89 ± 0.05 after MD (*p* = 0.001, *t*-test), while V1-activation was not different after non-deprived (contralateral) eye stimulation (noMD/MD: 2.37 ± 0.10/2.41 ± 0.37; *p* = 0.92, *t-test*). In 14dRW-PT mice, V1-activation after ipsilateral eye stimulation was 1.43 ± 0.37, and 1.08 ± 0.16 after MD (*p* = 0.33, *t-test*); after contralateral eye stimulation, V1-activation was 2.54 ± 0.63, and 2.65 ± 0.30 after MD (*p* = 0.85, *t-test*).

Together, these data clearly indicate that OD-plasticity is *not* impaired in V1 of the non-lesioned hemisphere after a stroke in the left S1, as has been shown previously for SC-raised C57Bl/6J mice (Greifzu et al., 2011).

### The Experience-Enabled Enhancement of the Optomotor Reflex of the Open Eye was also not Affected in 14dRW Mice After the Induction of a Cortical Lesion

Similar to RW-mice, mice transferred to a RW-cage directly after the induction of a cortical lesion in S1 (14dRW-mice) improved in both spatial frequency and contrast thresholds of the optomotor reflex of the open eye over the 7-day MD period.

#### Spatial Frequency Threshold

**Figure 9:**
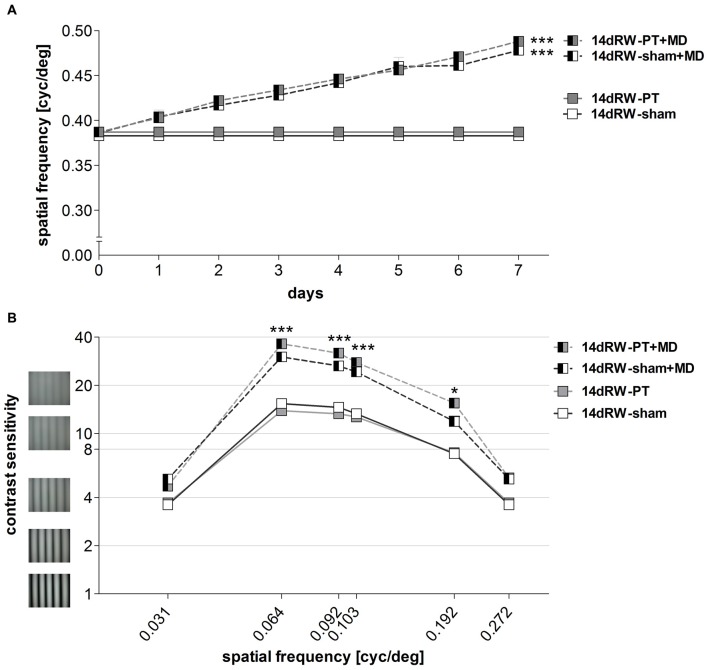
**Experience-enabled improvements of the optomotor reflex where not affected in 14dRW mice after cortical stroke in S1. (A)** The spatial frequency threshold of the optomotor reflex in cyc/deg plotted as a function of days. **(B)** Contrast sensitivity thresholds of optomotor reflex measured on 7th day of MD/noMD in six different spatial frequencies. All the groups without MD are illustrated as boxes and with MD as half-filled boxes. PT-groups are presented in gray color and sham groups in white. *p < 0.05, ***p < 0.001.

The highest spatial frequency that elicited an optomotor reflex increased in both non/PT-lesioned 14dRW-mice from 0.38 ± 0.003/0.39 ± 0.001 cyc/deg before MD to 0.48 ± 0.01/0.49 ± 0.005 cyc/deg after 7 days of MD (*n* = 3/7; *p* < 0.001, *t*-test). The MD-induced increase of the optomotor thresholds of both groups was 27% and 28% and thus was indistinguishable between the groups (*p* > 0.05, ANOVA; Figure 9A).

#### Contrast Sensitivity Threshold

The lowest contrast of a grating that elicited an optomotor response through the open eye also decreased, i.e., the contrast sensitivity increased in both non- and PT-lesioned 14dRW-mice after MD: values on the 7th day were significantly higher compared to noMD-mice at four out of the six spatial frequencies tested (*p* < 0.001 for spatial frequencies 0.064/0.092/0.103 cyc/deg, and *p* < 0.01 for 0.192 cyc/deg; ANOVA, Figure 9B).

In detail, in 14dRW-sham/PT mice, contrast sensitivity thresholds of the open eye (at 0.031, 0.064, 0.092, 0.103, 0.192 and 0.272 cyc/deg) improved to 5.2 ± 0.2 (19% contrast)/4.7 ± 0.1 (22%), 30.1 ± 1.8 (3%)/36.3 ± 2.2 (3%), 26.4 ± 1.2 (4%)/31.8 ± 1.5 (3%), 24.3 ± 1.8 (4%)/27.8 ± 1.4 (4%), 11.9 ± 0.9 (9%)/15.6 ± 0.7 (7%) and 5.2 ± 0.2 (19%)/5.3 ± 0.2 (19%) on day 7 (*p* > 0.05, *p* < 0.001, *p* < 0.001, *p* < 0.001, *p* < 0.01, *p* > 0.05, compared to day 0, ANOVA). The experience-enabled increase in contrast sensitivity thresholds after MD was indistinguishable between non- and PT-lesioned 14dRW-mice at any spatial frequency (*p* > 0.05, ANONA). Without MD, values of both groups remained stable over the 7-day measuring period (*p* > 0.05, for every frequency, ANOVA).

### Amount of Running in 14dRW Mice During the MD/noMD Period

To test: (i) whether a cortical lesion in S1 will affect the ability of the mice to run in a RW; and also (ii) if the amount of running has an effect on OD-plasticity after PT, the turns of the RW were measured daily in 14dRW-mice after the PT or sham treatment. Since there was no significant increase or decrease of running amount over days after MD for both groups of 14dRW-mice (sham: *p* = 0.14; PT: *p* = 0.12, ANOVA), we calculated an average amount of turns per day and animal. The 14dRW-sham mice (*n* = 6) ran on average 6.7 ± 1.2 km per day corresponding to 16665 ± 3047 turns of the wheel, while the 14dRW-PT mice (*n* = 12) ran on average 4.9 ± 1.00 km per day corresponding to 12235 ± 2470 turns of the wheel. The running amount did not differ between the non- and PT-lesioned mice (*p* = 0.29, *t*-test), suggesting that the cortical lesion in S1 was *not* interfering with the ability of mice to run on a RW. Furthermore, there were no significant differences between the running amount of the non and PT-lesioned mice after MD (sham + MD/PT + MD: 4.9 ± 1.7/6.6 ± 1.2 km, *n* = 3/7; *p* = 0.427, *t-test*), and values without or with MD were indistinguishable for both 14dRW-sham and 14dRW-PT mice (sham/sham + MD: *p* = 0.14, *t-test*; PT/PT + MD: *p* = 0.12, *t-test*), suggesting that the MD was not interfering with running.

Finally, we tested whether the amount of running and the ODI of individual mice were correlated, i.e., whether more running might cause stronger OD-shifts. To this end, the ODI of every mouse was plotted against the kilometers per day that each mouse runs for all the animals that received an MD. Analysis of the data showed that there was no correlation between the individual running amount and the ODI (*p* = 0.66, *R^2^* = 0.00248, correlation test).

## Discussion

In the present study, we analyzed whether voluntary physical exercise is beneficial for rescuing plasticity after a cortical stroke, in addition to being crucial for preserving visual plasticity into adulthood. Our results provide clear evidence that this was the case: raising mice in SCs equipped with a RW prevented impairments of visual plasticity after a stroke in S1-in contrast to SC-mice (Greifzu et al., 2011; Pielecka-Fortuna et al., 2015b). In addition, we could demonstrate the therapeutic potential of running *after* stroke: just 14 days of voluntary running after stroke restored plasticity to SC-raised adult mice. Notably, visual plasticity was restored in both tested paradigms: compared to SC-mice without a RW, OD-plasticity was preserved/restored in V1 *and* the experience-enabled improvements of the optomotor reflex of the open eye were rescued in both long-term and short-term running adult mice.

Having access to a RW for all their life, our adult RW-PT-mice were protected against the plasticity impairments normally occurring in (even younger) SC-raised mice after a stroke in S1 or secondary motor cortex (M2; Greifzu et al., 2011; Pielecka-Fortuna et al., 2015a). Notably, OD-plasticity was preserved into adulthood, similar as in the non-lesioned control RW-animals. Thus, our RW-PT-mice reacted to the stroke like juvenile SC-mice or EE-mice (Greifzu et al., 2014). Physical exercise is known to have beneficial effects on overall well-being, improving cognition and delaying age-related memory decline in humans (Cotman et al., 2007; Hillman et al., 2008). Mice with access to a RW have increased cell proliferation, higher neuron survival and elevated levels of BDNF in the hippocampus (Kobilo et al., 2011; Mustroph et al., 2012). Many molecular factors that changed after EE are also altered after physical exercise (Vivar et al., 2013); e.g., increased levels of BDNF (Berchtold et al., 2005), NGF (Neeper et al., 1996), and IGF (Carro et al., 2000). Interestingly, exposure of rats to either EE or treadmill exercise reduced the susceptibility to cortical spreading depression (Santos-Monteiro et al., 2000; Lima et al., 2014), thereby possibly contributing to reduced plasticity-impairing effects of a cortical PT-lesion after free access to a RW.

Although many studies showed positive effects of physical exercise on the brain, only recently the effect of running on visual cortical activity and plasticity has been addressed. Locomotion increases firing rates in V1 (Niell and Stryker, 2010) and the lateral geniculate nucleus (Erisken et al., 2014), and enhancements of visual responses induced by locomotion are sufficient to promote recovery of visual function after long term MD (Kaneko and Stryker, 2014). Voluntary physical exercise also preserved OD-plasticity into adulthood and restored this plasticity in SC-raised adult mice (Kalogeraki et al., 2014). Additionally, the activity of a specific class of V1 interneurons that express vasoactive intestinal protein (VIP) is directly modulated by locomotion (Fu et al., 2014). A neural circuit underlying these effects was proposed, in which the specific recruitment of VIP-cells by locomotion directly modulated V1-activity through a disinhibitory mechanism (Fu et al., 2014; Lee et al., 2014). It was also shown that providing adult rats with RWs for 3 weeks caused a decrease in GABA-release (Baroncelli et al., 2012). Extended running experience by raising mice in SCs with RWs may thus “chronically” reduce inhibitory drive onto pyramidal cells and thus promote cortical plasticity.

In addition to these plasticity-promoting effects of physical exercise on a healthy brain, studies also pointed out the beneficial effects of physical exercise *after* brain lesions. RW-exercise increased the number of newborn hippocampal neurons after PT-stroke in mice and improved spatiotemporal learning (Geibig et al., 2012). Mice with middle cerebral artery occlusion (MCAO) showed long-term functional and cognitive improvements after running (Gertz et al., 2006). Physical exercise was also neuroprotective: running 2–3 weeks before an MCAO-stroke reduced cerebral infarct size and sensory-motor deficits in rodents (Wang et al., 2001; Endres et al., 2003).

One possibility how physical exercise may influence OD-plasticity after stroke is through remodeling of the extracellular matrix by matrix metalloproteinase (MMP) activity. A recent study reported elevated levels of MMP9 after mild treadmill exercise in rats (Nishijima et al., 2015), demonstrating that running influences MMP activity. MMPs play an important role for plasticity in both the healthy and diseased brain (Huntley, 2012). MMP-activity is a crucial component of OD-plasticity in the healthy juvenile and adult brain (Spolidoro et al., 2012; Pielecka-Fortuna et al., 2015b). After cerebral ischemia, the expression of some MMPs is increased (Gasche et al., 1999; Heo et al., 1999; Montaner et al., 2001; Rosell et al., 2006). Inhibition of MMPs with a broad-spectrum MMP-inhibitor immediately before a stroke partially rescued experience-dependent barrel cortex plasticity in mice (Cybulska-Klosowicz et al., 2011). Recently, we revealed that an optimal level of MMP-activity is crucial for adult visual cortex plasticity in the healthy and stroke-affected brain (Pielecka-Fortuna et al., 2015a): in healthy mice, inhibition of MMP-activity with a broad spectrum inhibitor during the 7-day-MD-period resulted in lost OD-plasticity; in contrast, lowering stroke-induced elevated levels of MMPs with an inhibitor *after* stroke rescued OD-plasticity.

Stroke also affects the balance between excitation and inhibition in the affected neuronal network (Carmichael, 2006). High levels of the neurotransmitter glutamate after ischemic stroke lead to excitotoxicity and neuronal cell death (Lai et al., 2014). Additionally, a lesion in V1 caused reduced basal GABAergic transmission measured away from the lesion border (Imbrosci et al., 2015). It seems that changes in both glutamatergic and GABAergic transmission after stroke can lead to negative consequences and thus may interfere with plasticity. EE-raising not only preserved a low GABAergic inhibition and juvenile OD-plasticity into adulthood, it also protected adult mice from stroke induced impairments of V1-plasticity after an S1-lesion (Greifzu et al., 2014). Physical exercise influences many neurotransmitter systems in the brain, including glutamatergic (Farmer et al., 2004; Vasuta et al., 2007; Lou et al., 2008) and GABAergic (Molteni et al., 2002). Altogether, these studies imply that a lower inhibitory tone after running may protect V1 from plasticity impairments after an S1-lesion. Likewise, short-term dark exposure reduced the inhibitory tone in V1 (He et al., 2006) and preserved OD-plasticity after an S1-lesion (Stodieck et al., 2014). In summary, exposing mice to conditions which reduce the inhibitory tone in V1, protected them against stroke-induced impairments of visual plasticity. Interestingly, modification of excitatory circuits was recently shown to be also beneficial for preserving plasticity after stroke: OD-plasticity in V1 was preserved after an S1-stroke in mice lacking postsynaptic density protein-95 (Greifzu et al., 2016b), a signaling scaffold protein present at mature excitatory synapses necessary for synaptic maturation (Huang et al., 2015).

Furthermore, the basic parameters of spatial vision and their experience-enabled improvements were tested using optomotry (Prusky et al., 2004). After an S1-lesion, RW-mice showed significant enhancements in both the spatial frequency and contrast sensitivity thresholds of the optomotor reflex of the open eye after MD. In contrast, an S1-lesion in adult SC-raised mice without a RW abolished any threshold improvements (Greifzu et al., 2011). These improvements were rescued by either anti-inflammatory treatment or by waiting for 14 days between lesion and MD (Greifzu et al., 2011). In EE-raised mice, increases in optomotor thresholds were also detected after stroke, but the rescue was only partial (Greifzu et al., 2014). In contrast, in the present study, both short-term and long-term running fully restored the optomotor threshold increases, and the final values were similar to those of *non-lesioned* SC- (Prusky et al., 2004) or RW-mice (Kalogeraki et al., 2014). Whether running has an anti-inflammatory effect remains to be determined.

Quantitative analyses showed no significant differences in lesion localization, length, depth and volume between lifelong and 14dRW-mice, only a smaller diameter in the 14dRW-mice. There was, however, no correlation between lesion volume and the ODI after MD, in agreement with previous observations (Greifzu et al., 2011). Moreover, even a smaller and three times more distant lesion in M2, can affect OD-plasticity in V1 (Pielecka-Fortuna et al., 2015b). Thus, the smaller lesion diameter in 14dRW-mice is most probably not causing the preserved plasticity (lesion volume was similar to the long-term RW-group). In summary, the restoration of OD-plasticity in both groups of RW-mice is most likely due to their voluntary physical exercise, and not caused by any differences in lesion location or volume.

Summarizing, our data show that voluntary physical exercise with RWs not only preserved visual plasticity into adulthood, but also restored OD-plasticity in adult mice after stroke. Additionally, physical exercise in stroke-lesioned mice restored the experience-enabled improvements of the spatial frequency and contrast sensitivity thresholds of the optomotor reflex. More interestingly, even short-term running for just 14 days *after* a stroke was already effective in restoring both OD-plasticity and improvements of the optomotor reflex in adult SC-raised mice. Interestingly, the magnitude of the induced OD-shifts and of the optomotor threshold improvements were indistinguishable between the long-term and short-term running groups. This demonstrates that voluntary physical exercise can protect from plasticity impairments caused by a cortical stroke, *and* is effective even if the exercise starts only in the post-stroke period. Thus, the present data advertise voluntary physical exercise as a promising candidate for post-stroke plasticity promoting therapies.

## Author Contributions

Conception and design of the work (EK, JP-F, SL), acquisition of data (EK, JP-F, JMH), analysis and interpretation of data (EK, JP-F, JMH, SL), drafting and revising of the article (EK, JP-F, SL). All authors approved the final version of the manuscript.

## Funding

This work was supported by the Federal Ministry of Education and Research, Germany, Grants 01GQ0921 (to EK and JP-F) and 01GQ0810 (to SL), by an Alexander von Humboldt Research Fellowship for Postdoctoral Researchers (to JP-F) and by grants of the Deutsche Forschungsgemeinschaft through the Collaborative Research Center 889 “Cellular Mechanisms of Sensory Processing” to Siegrid Löwel (Project B5).

## Conflict of Interest Statement

The authors declare that the research was conducted in the absence of any commercial or financial relationships that could be construed as a potential conflict of interest.
